# Editorial: Transcription factors in cardiovascular development and remodeling

**DOI:** 10.3389/fcell.2023.1225947

**Published:** 2023-06-06

**Authors:** Liudan Zhao, Yu Yu

**Affiliations:** Institute for Development and Regenerative Cardiovascular Medicine, Xinhua Hospital, School of Medicine, Shanghai Jiao Tong University, Shanghai, China

**Keywords:** transcription factors, cardiovascular disease, developmental biology, remodeling, signaling

Cardiovascular diseases (CVDs), involving diverse pathological conditions of the heart and the vascular system, are the predominant causes of death worldwide (Tsao et al., 2023). In childhood, CVDs are mainly manifested as congenital heart diseases (CHDs), which arise from abnormal or incomplete formation of cardiovascular development and affect at least 2% of newborns (Dolk et al., 2011). According to the estimation, 13.3 million people worldwide had CHDs in 2019 (Roth et al., 2020), with a high likelihood of developing physical and mental health complications across the life span. Adults are more likely to develop chronic cardiovascular disease, which includes atherosclerosis, thrombosis, myocardial infarction, hypertension, pulmonary arterial hypertension (PH), and cardiomyopathy. PH and cardiomyopathy can concomitantly appear with CHD in children. CVDs affects 48.6% of adults with ≥20 years of age (127.9 million in 2020) and increases with age in both male and female (Akinbami et al., 2022). Given that CVDs are diseases throughout the lifespan, immense health and economic burdens have been produced and it is therefore important to explore the underlying mechanisms that contribute to disease development.

Dysfunctions of cellular signaling transduction and gene expression have been widely recognized as critical molecular mechanisms in the development of CVDs (Ghigo et al., 2017). Among numerous signaling molecules, transcription factors (TFs) are essential for the development and maintenance of the cardiovascular system. The key role of a TF is to recruit transcriptional regulatory components to certain genomic loci to modulate cardiovascular genes expression and thus control important morphogenetic steps (Ptashne and Gann, 1997). Most of the inherited forms of CVDs are caused by the mutations in cardiovascular TF genes. Congenital heart defects, for example, are commonly associated with loss-of-function mutations in related TFs (Epstein and Buck, 2000). Moreover, the function of TFs in cardiovascular remodeling also has become a research hotspot, revealing novel therapeutic approaches against chronic CVDs of adults (Potente et al., 2005; Li et al., 2017; Dittrich et al., 2021). Therefore, a deeper understanding of the transcriptional regulatory mechanisms controlling cardiovascular genes crosstalks will be critical for deciphering the molecular etiology of CVDs, as well as for helping to design more powerful and specific regenerative therapies for CVDs.

The aim of this Research Topic is to shed light on recent progress and trends in the function of transcription factors in cardiovascular development and remodeling. It currently includes two research articles and two reviews from 41 authors, covering high-incidence CVDs and cutting-edge bioinformatics methods and presenting potential therapeutic genetic targets. The collection encompasses different fields of investigations as further detailed by the sections below.

Ventricular septal defect (VSD) is defined as an interruption in the interventricular septum formation during cardiac morphogenesis, accounting for 40% of all CHDs (Penny and Vick, 2011). In past 10 years, a number of gene disruptions have been shown to cause complex VSD, however functional non-coding SNPs (cis-regulatory SNPs) have not been examined systematically. Jin et al. performed an exome-wide association analysis using whole-excome sequencing data of 80 patients (includes tetralogy of fallot and pulmonary atresia with VSD) and 100 controls in Chinese children and identified 93 low-frequency noncoding SNPs associated with complex VSD risk. Specifically, rs2279658 might cause abnormal transcription of COQ2 and FAM175A in the heart, which contributes to complex VSD predisposition. These findings highlight the importance of cis-regulatory SNPs in the pathogenesis of complex VSD and broaden our knowledge of this disease.

PH is a rare but lethal disease characterized by remodeling of the pulmonary arteries, increased vascular resistance, ultimately resulted in right heart failure and even death (Walters et al., 2023). It can concomitantly appear with CHD in children and also occur in adult. Numerous studies have uncovered novel genetic risk variants involved in the pathogenesis of PH, such as hypoxia-inducible factors family. Endothelial PAS domain-containing protein 1 (EPAS1), also referred to hypoxia-inducible factor 2 alpha (HIF-2α), is mainly expressed in the endothelial cell, while its mechanism in PH still remains largely unknown. The molecular structure, biological function and regulatory network of EPAS1 in PH were presented in detail by Wang et al., which provided theoretical support for the possible novel target for future PH intervention. Additionally, the work of Yang et al. studied the role of transcription factors in various PH-related cellular mechanisms including in pulmonary arterial smooth muscle cells, pulmonary arterial endothelial cells, pulmonary arterial fibroblasts and inflammatory cells. These reviews improved the understanding of particularly interactions among transcription factors mediated cellular signaling pathways and identified novel therapies for PH.

With the increasing scale of high-throughput expression data, pathway analysis has been widely applied in the field of CVDs, which uncovered several potential mechanisms of CVDs. MicroRNAs (miRNA) is known to cause gene silencing by binding to mRNA, while competitive endogenous RNA (ceRNA) can regulate gene expression by competitively binding to miRNA. CeRNA has been shown to play the significant role in cellular biology (Song et al., 2017), in which several genes can interact with others. However, there is a lack of comprehensive analyses concerning the ceRNA-mediated pathway–pathway crosstalk in CVDs. Therefore, Song et al. utilized 21 gene expression datasets to generate a landscape of the ceRNA-mediated pathway–pathway crosstalk of eight major CVDs among adults and children. It elaborated the potential molecular regulatory mechanisms of ceRNA interaction in pathway–pathway crosstalk, which innovated the current identification methods of the pathway crosstalk.

In summary, this Research Topic gathered a broad range of high-quality articles demonstrating the latest advances of transcription factors in cardiovascular development and remodeling. More cardiovascular research beyond the four articles is needed, and future topics will address specific areas in pediatrics and adult heart research field. We welcome all articles of related research topics and hope that this Research Topic will constantly serve to inspire the scientific community and medical treatment by providing new strategy for understanding biological process and human diseases.
